# The Efficacy and Safety of Surgical Treatment for Patients With Tuberculosis Destroyed Lung With or Without Chronic Pulmonary Aspergillosis

**DOI:** 10.1007/s00268-021-05969-w

**Published:** 2021-02-08

**Authors:** Hongyun Ruan, Changfan Gong, Jinxiang Wang

**Affiliations:** 1 Cardiopulmonary Function Department, Beijing Chest Hospital, Capital Medical University, Beijing, China; 2 Department of Thoracic Surgery, Beijing Chest Hospital, Capital Medical University, Beijing, China; 3 Department of Pulmonary and Critical Care Medicine, Beijing Luhe Hospital, Capital Medical University, Beijing, China

## Abstract

**Background:**

To evaluate the efficacy and safety of surgical treatment of tuberculosis destroyed lung (TDL), and the influence of chronic pulmonary aspergillosis (CPA) on the outcomes of surgical treatment of TDL.

**Methods:**

We performed a retrospective analysis of 113 patients with TDL who underwent surgical treatment from January 2005 to December 2019. Among them, 30 of these cases were complicated with CPA. The patients were divided into two groups: TDL group and TDL + CPA group. We analyzed the effectiveness and safety of surgical treatment of TDL, and further compared the effectiveness and safety of surgical treatment of TDL with or withoutthe presence of CPA.

**Results:**

The TDL + CPA group had a significantly higher age (*P*=0.003), symptoms of hemoptysis (*P*=0.000), and a higher proportion of patients with preoperative serum albumin <30 g/L (*P*=0.014) as compared with TDL group. For all enrolled patients, the incidence of severe postoperative complications was 12.4% (14/113) and the postoperative mortality within 30 days after discharge was 4.4% (5/113). 86.7% (98/113) of the patients recovered and discharged, the incidence of severe postoperative complications in the TDA + CPA group was higher than that of TDL group (23.3% vs 8.4%, *P* = 0.034), although there was no difference in mortality between the two groups (*P* = 1.000). A binary logistic regression analysis showed that the independent risk factors for severe postoperative complications were male (OR 25.24, 95% CI 2.31–275.64; *P* = 0.008) and age ≥ 40 years (OR 10.34, 95% CI 1.56–68.65; *P* = 0.016).

**Conclusion:**

Surgical treatment for patients with TDL is effective with an acceptable mortality rate whether or not the disease is complicated with CPA. The independent risk factors identified for severe postoperative complications in patients with TDL were male and ≥ 40 years old. It implies that when treating patients with TDA + CPA, particular attention should be paid to these patients who have these independent risk factors to avoid a poor outcome.

## Introduction

Chronic lung infection can result in destroyed lung, characterized by diffuse lung structural damage, is more common in developing countries. Tuberculosis is the most common cause of destroyed lung [1, 2]. The prevalence of CPA ranged from 6.3 to 13.7% in patients previously or currently being treated for tuberculosis [3], residual cavity is more susceptible to complications of chronic pulmonary as per gillosis (CPA) [4, 5]. The most common symptom of pulmonary tuberculosis and CPA is hemoptysis [6–9], and massive hemoptysis is an indication of surgical treatment [2, 10–12]. Surgical treatment is also a necessary and important means for treating patients with multidrug resistant tuberculosis (MDR-TB) [11, 13, 14] or poor response to antifungal therapy [15]. Many studies have shown that the surgical treatment of tuberculosis destroyed lung (TDL) or CPA is effective, with acceptable postoperative complications and mortality. Therefore, surgical treatment has become an important treatment option for this population [6, 7, 9, 16–19].

The main purpose of surgical treatment of TDL is to solve life-threatening complications and improve the quality of life [20]. Surgical treatment can also help treat MDR-TB and prevent recurrence of the disease [11, 13]. Antifungal therapy is the main treatment of CPA [15]. However, antifungal therapy requires a long course and has a low success rate [21]. If there is life-threatening hemoptysis, or a poor response to antifungal therapy, surgical treatment should be considered [15]. Moreover, surgical resection may be a better option than long-term antifungal therapy if patients cannot afford the cost of long-term treatment, drug treatment failure or intolerance to long-term oral antifungal therapy [15, 22]. If patients have both TDL and CPA, the treatment is even more complicated. There have been few studies in this area, but one study involving some CPA patients complicated with TBL reported that surgical treatment can benefit patients with CPA [7].

In order to evaluate the efficacy and safety of surgical treatment of TDLwith or without CPA, we retrospectively analyzed 113 patients who underwent surgical treatment for TDL in Beijing Chest Hospital from January 2005 to December 2019; thirty of the included cases were complicated with CPA.

## Materials and methods

### Study design and objectives

From January 2005 to December 2019, all patients with TDL who underwent surgical treatment in Beijing Chest Hospital were enrolled in this study. Among them, 30 patients were confirmed as *Aspergillus* infection by histopathology. This study was approved by the Ethics Committee of Beijing Chest Hospital affiliated to Capital Medical University (Ethics number: clinical study 2018 (43)). The requirement for informed consent from the patients was waived by the ethical review board.

The patients were divided into two groups: TDL group and TDL + CPA group. General clinical data were collected, including gender, age, smoking status, alcohol abuse, body mass index, comorbidities, symptoms (hemoptysis, cough, fever, dyspnea, and chest pain), preoperative lung function, routine laboratory examination, CPA classification, intraoperative blood loss, severe postoperative complications, death (postoperative during hospitalization and within 30 days after discharge), and the cause of death. Severe postoperative complications included severe respiratory failure due to infection requiring invasive mechanical ventilation for more than 72 h, heart failure, empyema, bronchopleural fistula, and reoperation due to massive postoperative bleeding.

The diagnosis of CPA was based on the criteria of ERS and ESCMID [22]: one or more cavities with or without a fungal ball present or nodules on thoracic imaging; direct evidence of *Aspergillus* infection (microscopy or culture from biopsy) or, an immunological response to *Aspergillus spp*. and exclusion of alternative diagnoses, all present for at least 3 months.The diagnosis of CPA included simple aspergilloma, chronic cavitary pulmonary aspergillosis (CCPA), chronic fibrosing pulmonary aspergillosis (CFPA), *Aspergillus*nodule and subacute invasive aspergillosis (SAIA).

Jaeger Master Screen PFT was used to measure lung function. Lung diffusion capacity for carbon monoxide was measured using single breath method. Lung function data include: forced expiratory volume in one second of predicted (FEV_1_% pred); forced vital capacity of predicted (FVC% pred); the ratio of forced expiratory volume in one second to forced vital capacity (FEV_1_/FVC); vital capacity of predicted (VC% pred); maximal minute ventilation of predicted (MMV% pred); inspiratory capacity of predicted (IC% pred); functional residual volume of predicted (FRC% pred); residual volume of predicted (RV% pred); total lung capacity of predicted (TLC% pred); the ratio of total lung capacity to residual volume (RV/TLC); and lung diffusion capacity for carbon monoxide of predicted (D_L_CO% pred).

The surgical indication of TDL was that the lesions were confined to the ipsilateral lung with the following conditions were met: TDL and CPA were located in the ipsilateral lung; massive hemoptysis; persistent positive of sputum smear and/or culture of *Mycobacterium tuberculosis*; antituberculosis treatment was ineffective or intolerable; recurrent infection of TDL. Surgical contraindications were as follows: respiratory failure in preoperative blood gas analysis; serious comorbidities; bilateral involvement of lesions. Whether or not to take surgical treatment for TDL patients was discussed and decided by a team of experts from radiology, thoracic surgery and anesthesiology.

The aim of our study was to evaluate the efficacy and safety of surgical treatment of TDL and by comparative analysis the efficacy and safety of surgical treatment of TDL complicated with CPA.

## Statistical analysis

SPSS version 21.0 for Windows software (SPSS Inc.,Chicago, IL, USA) was used for data management and statistical analysis. Continuous variables data were expressed as the mean ± SD, whereas categorical data were presented as a number or percentage. Continuous variables data were compared using the parametric unpaired, two-independent-group Student's t-test. Binary logistic regression analysis was used to assess the following risk factorsfor severe postoperative complications: gender, age ≥40 years [23], smoking, comorbidities, CPA, low body mass index (<18.5 kg/m^2^), anemia (hemoglobin <90 g/L) and low serum albumin (<30 g/L) before operation. We calculated the risk factors for odds ratio (OR) and95% for confidence interval (CI). The Pearson Chi-square test or continuous correction Chi-square test was used to compare the counting data and *P*<0.05 was considered statistically significant.

### Result

A total of 113 patients with TDL who underwent surgical treatment were enrolled, 51 males and 62 females, with a mean age of 39.2 ± 13.6 years. Thirty patients had TDL complicated with CPA, 17 males and 13 females with a mean age of 46.2 ± 15.3 years. Four cases in CPA group and nine cases in TDL + CPA group did not receive anti-tuberculosis therapy before operation. For 30 patients of TDL complicated with CPA, three cases received itraconazole and 27 cases received voriconazole for antifungal treatment during perioperative period. The distribution of CPA types included, nine cases of CCPA, two cases of CFPA, three cases of simple aspergilloma, five cases of *Aspergillus* nodule and four cases of SAIA. In the TDL group, three cases were complicated with nontuberculous mycobacterium infection (Table 1).

**Table 1:** Baseline clinical characteristics of TDL with or without CPA

Characteristics	TDL group (*n* = 83)	TDL + CPA group (*n* = 30)	*P* Value
*Sex*			
Male	34 (41.0)	17 (56.7)	0.139
Age	36.7 ± 12.0	46.2 ± 15.3	0.003
BMI < 18.5 kg/m^2^	13 (15.7)	7 (23.3)	0.346
Smoking status: Smoking	11 (13.3)	5 (16.7)	0.646
Comorbidities*	6 (7.2)	3 (10.0)	0.931
*Symptom*			
Hemoptysis	20 (24.1)	19 (63.3)	0.000
Cough	39 (47.0)	11 (36.7)	0.329
Fever	7 (8.4)	4 (13.3)	0.677
Dyspnea	28 (33.7)	5 (16.7)	0.078
Hemoglobin < 90 g/L	7 (8.4)	3 (10.0)	1.000
Serumalbumin < 30 g/L	3 (3.6)	6 (20.0)	0.014
MDR-TB	9 (10.8)	4 (13.3)	0.974
*Lung function*			
FEV_1_ (% pred)	54.2 ± 15.1	67.8 ± 24.3	0.035
FVC (% pred)	62.9 ± 15.6	70.7 ± 22.2	0.173
FEV_1_/FVC	74.0 ± 12.5	79.1 ± 13.8	0.139
RV (% pred)	123.1 ± 37.2	115.8 ± 38.8	0.470
TLC (% pred)	79.8 ± 17.6	84.5 ± 17.0	0.311
RV/TLC	150.9 ± 28.1	132.3 ± 41.7	0.088
IC (% pred)	67.9 ± 27.2	64.6 ± 26.5	0.643
VC (% pred)	61.5 ± 15.2	70.2 ± 20.9	0.110
MMV (% pred)	53.7 ± 14.7	66.6 ± 28.4	0.078
D_L_CO (% pred)	58.5 ± 15.7	74.4 ± 34.2	0.070
*Arterial blood gas analysis*			
pH value	7.40 ± 0.04	7.41 ± 0.03	0.650
PaCO_2_	42.8 ± 4.0	43.4 ± 3.8	0.556
PaO_2_	104.6 ± 37.8	101.9 ± 28.2	0.810

Data are presented as mean ± SD or number (%)

*TDL* tuberculosis destroyed lung; *CPA* chronic pulmonary aspergillosis; *FEV*_1_ (% pred), forced expiratory volume in one second of predicted; *FVC* (% pred) forced vital capacity of predicted; *FEV*_*1*_*/FVC* the ratio of forced expiratory volume in one second to forced vital capacity; *RV* (% pred), residual volume of predicted; *TLC* (% pred), total lung capacity of predicted; *RV/TLC* the ratio of total lung capacity to residual volume; *IC* (% pred), inspiratory capacity of predicted; *VC* (% pred), vital capacity of predicted; *MMV* (% pred), maximal minute ventilation of predicted; *D*_*L*_*CO* (% pred), lung diffusion capacity of predicted

*Comorbidities in TDL group included 4 cases of diabetes, 1 case of congenital atrial septal defect and 1 case of ankylosing spondylitis; comorbidities in TDL + CPA group included 1 case of hypertension and 2 cases of diabetes

We found the mean age was higher (46.2 ± 15.3 years vs. 36.7 ± 12.0, *P* = 0.003), and the symptom of hemoptysis was more common (*P* = 0.000) in TDL + CPA group as compared to the TDL group; other symptoms of cough, expectoration, fever and dyspnea had no observable differences between the two groups. We also observed that FEV_1_% pred was higher in the TDL + CPA group than in the TDL group (67.8 ± 24.3 vs. 54.2 ± 15.1, *P* = 0.035), but no statistical difference (*P* > 0.05) between the two groups in FVC% pred, FEV_1_/FVC, MMV% pred, VC% pred, IC% pred, RV% pred, RV/TLC, TLC% pred and D_L_CO% pred (Table 1).

The proportion of patients with preoperative serum albumin <30 g/L in the TDL + CPA group was higher than that in the TDL group (*P*=0.014). There was no statistical difference (*P*>0.05) between the two groups for body mass index, smoking status, main comorbidities, preoperative anemia (hemoglobin<90 g/L), preoperative arterial blood gas analysis index and the incidence of MDR-TB. Three cases of nontuberculous mycobacterium infection in the TDL group (Table 1).

Among 113 patients, 34 patients underwent lobectomy and 79 underwent pneumonectomy. There was no recurrence of massive hemoptysis, the incidence of severe postoperative complications was 12.4% (14/113), and the postoperative mortality within 30 days after discharge was 4.4% (5/113), 86.7% (98/113) of the patients recovered gradually and discharged. The incidence of severe postoperative complications in TDL + CPA group was significantly higher than that in the TDL group (23.3% vs. 8.4%, *P*=0.034), although there was nodifference in the amount of intraoperative blood loss, the postoperative mortality within 30 days after discharge, and the postoperative length of hospital stay (Table 2).

**Table 2:** Clinical outcomes of TDL with or without CPA

Characteristics	TDL group (*n* = 83)	TDL + CPA group (*n* = 30)	*P* Value
Intraoperative blood loss, ml	871 ± 796	1393 ± 1575	0.091
Severe postoperative complications	7 (8.4)	7 (23.3)	0.034
Severe respiratory failure	2	4	
Heart failure	1	0	
Empyema	0	1	
Bronchopleural fistula	0	1	
Massive postoperativebleeding	4	1	
Postoperative hospital stay, days	29.5 ± 22.1	37.5 ± 25.5	0.106
Death after operation, during hospitalization or within 30 days of discharge	4 (4.8)	1 (3.3)	1.000

Data are presented as mean ± SD or number (%)

*TDL* tuberculosis destroyed lung; *CPA* chronic pulmonary aspergillosis

In the TDL group, seven cases had severe postoperative complications, including four cases of massive postoperative bleeding, one case of heart failure, two cases of severe respiratory failure due to recurrent pulmonary infection. Four cases died postoperative or within 30 days following discharge, one case died of severe respiratory failure and three cases died of massive hemorrhage (Table 2).

In TDL + CPA group, seven cases had severe postoperative complications, including one case of empyema, one case of massive postoperative bleeding, four cases of severe respiratory failure, and 1 case of bronchopleural fistula. One patient died of bronchopleural fistula complications (Table 2).

Reoperation for severe postoperative complications: In TDL group, one patient with massive postoperative bleeding survived after reoperation, and the other three patients with massive postoperative bleeding were critically ill and did not have the conditions for reoperation and died. In TDL + CPA group, the patients with massive postoperative bleeding or bronchopleural fistula were reoperated, and the patient with bronchopleural fistula died.

The binary logistic regression analysis showed that the independent risk factors for severe postoperative complications were male gender (OR 25.24, 95% CI 2.31–275.64; *P* = 0.008) and age ≥ 40 years (OR 10.34, 95% CI 1.56–68.65; *P* = 0.016) (Table 3).

**Table 3:** The Binary logistic regression analysis for risk factors of severe postoperative complications in patients with TDL

Characteristics	≥40 years old	Male	BMI <18.5 kg/m^2^	Smoking	Hemoglobin <90 g/L	Albumin <30 g/L	CPA	Comorbidities
OR	10.342	25.236	6.136	0.612	7.300	0.754	2.509	1.043
95% CI	1.558–68.654	2.310–275.643	0.856–43.977	0.105–3.636	0.808–65.973	0.068–8.324	0.539–11.671	0.097–11.209
*P* Value	0.016	0.008	0.071	0.585	0.077	0.818	0.241	0.972

*BMI* body mass index; *CPA* chronic pulmonary aspergillosis

## Discussion

Destroyed lung is an irreversible disease defined by radiology with the physiological characteristic of decreased ventilation/perfusion ratio, with the primary cause from pulmonary tuberculosis [2], that is common in countries with underdeveloped health systems [24]. In Asia and Africa, the co-infection rate of *Aspergillus* and tuberculosis is 15.4%, and it is higher in individuals above 40 years old [25]. Male tuberculosis patients are more likely to be infected with fungi, which may be related to high exposure to the surrounding environment [26]. In our study, the rate of dyspnea had a trend higher in TDL group than TDL + CPA group before surgery (*P* = 0.078), and the blood gas results of the two groups were similar, which may be due to the fact that blood gas analysis mainly reflects respiratory function, which is only one of the important factors affecting dyspnea. There was no gender difference in patients with TDL whether or not complicated with CPA (*P*=0.139), but the age of patients in the TDL + CPA group was higher than that in the TDL group, indicating that elderly patients with TDL are more likely to have fungal infection, consistent with the conclusion of Hosseini et al. [25].

Surgical treatment of destroyed lung can solve life-threatening complications and improve the quality of life for affected patients [20]. It has been proven that the postoperative complications and mortality of surgical treatment of patients with TDL or CPA are acceptable [6, 7, 9, 16–18]. Indications for surgical treatment of destroyed lung include massive hemoptysis, empyema, secondary fungal infection and respiratory failure that have affected the quality of life [2]. When TDL is complicated with MDR-TB, surgical resection is also helpful for negative conversion of MDR-TB and preventing recurrence [11, 13]. In our results, the incidence of severe complications and mortality were 12.4% and 4.4%, respectively, similar to those reported in the literature [2, 17–19]. Although the incidence of severe postoperative complications was higher in the TDL + CPA group than in the TDL group, there was no difference in the mortality between the two groups postoperatively during hospitalization or within 30 days after discharge. Therefore, surgical treatment of TDL complicated with CPA was also effective with an acceptable mortality rate.

Patients with pulmonary tuberculosis are susceptible to CPA, and antifungal drugs are the first choice for the treatment [15, 27]. However, the course of antifungal therapy is long and the success rate is low [21]. Moreover, many CPA patients live in low- and middle-income countries, where surgical resection may be better than long-term antifungal therapy due to poor economic conditions, potential drug treatment failure and intolerance to long-term oral antifungal therapy [15, 22]. Our study showed that even if the TDL is complicated with CPA or not, surgical treatment is a safe and effective option. Although for patients with ineffective or intolerable medical treatment, as well as patients with life-threatening hemoptysis, especially for the TDL patients with CPA, surgical treatment is a good choice. However, there were some serious complications and deaths after operation, which should be paid attention to and treated actively.

It has been reported that hypoalbuminemia increases the risk of postoperative complications of TDL [2] and is associated with increased mortality in patients with CPA [28]. Our study showed that the independent risk factors of severe postoperative complications in patients with TDL are male gender and an age ≥40 years old, that may be related to the susceptibility of male and elderly patients to CPA. However, we did not find that hypoalbuminemia was associated with an increase in severe postoperative complications.

This study had several limitations. Firstly, for the retrospective study, the follow-up time is short, no follow-up of lung function. Therefore, the recovery of postoperative pulmonary function, whether there is chronic respiratory failure and the need for home oxygen therapy, and the postoperative quality of life are not clear, which need to be further studied. Secondly, due to the small number of cases, especially the small number of CPA cases, we were unable to carry out a subgroup analysis on CPA, nor did we conduct subgroup analysis on specific surgical procedures. Finally, owing to the large time span of included cases, there are differences in medical practice and technologies across this time span that would certainly impact the results. However, this is inevitable in any retrospective study.

In conclusion, surgical resection is effective in the treatment of TDL with an acceptable mortality rate whether or not it is complicated with CPA. The independent risk factors for severe postoperative complications in patients with TDL were male and ≥40 years old. The increased risk of postoperative severe complications in patients with TDL complicated with CPA deserves attention.
